# Beyond Digital Twins: Phygital Twins for Neuroergonomics in Human-Robot Interaction

**DOI:** 10.3389/fnbot.2022.913605

**Published:** 2022-06-28

**Authors:** Giacinto Barresi, Claudio Pacchierotti, Matteo Laffranchi, Lorenzo De Michieli

**Affiliations:** ^1^Rehab Technologies, Istituto Italiano di Tecnologia, Genoa, Italy; ^2^CNRS, Univ Rennes, Inria, IRISA, Rennes, France

**Keywords:** neuroergonomics, robotics, human-robot interaction, Digital Twin, Phygital Twin, human-machine interfaces, neurorobotics

## Introduction

Among the most recent enabling technologies, Digital Twins (DTs) emerge as data-intensive network-based computing solutions in multiple domains—from Industry 4.0 to Connected Health (Pires et al., 2019; Bagaria et al., 2020; Juarez et al., 2021; Phanden et al., 2021). A DT works as a virtual system for replicating, monitoring, predicting, and improving the processes and the features of a physical system—the Physical Twin (PT), connected in real-time with its DT (Grieves and Vickers, 2017; Kaur et al., 2020; Mourtzis et al., 2021; Volkov et al., 2021). Such a technology, based on advances in fields like the Internet of Things (IoT) and machine learning (Kaur et al., 2020), proposes novel ways to face the issues of complex systems as in Human-Robot Interaction (HRI) (Pairet et al., 2019) domains.

This position paper aims at proposing a physical-digital twinning approach to improve the understanding and the management of the PT in contexts of HRI according to the interdisciplinary perspective of neuroergonomics (Parasuraman, 2003; Frederic et al., 2020).

## Approaching and Adopting Digital Twins

The DT definition is still an object of debate, and reaching one could be a necessary step for efficiently managing its technical requirements in terms of computing and connectivity (Shafto et al., 2012; Haag and Anderl, 2018; Jones et al., 2020; Kuehner et al., 2021; Singh et al., 2021; Botín-Sanabria et al., 2022; Wang D. et al., 2022). However, we can ignite our discussion by considering how Fuller et al. (2020) highlighted that a DT is not just a digital model or an offline simulation of a physical object. Nor does a DT correspond to a digital shadow, depicting the real-time states and changes of a PT that can just be manually modified. The changes in a DT automatically mirror and affect the status of its PT: the data flows bi-directionally (Van der Valk et al., 2020) and in real time between twins in digital and physical worlds, possibly without any human intervention (Liu et al., 2022) through the DT-driven control of an actuated PT. However, a DT is typically “played” by experts like managers, engineers, and designers as a complex interactive simulation to predict future issues in the PT according to its past and current behavior (Semeraro et al., 2021). This leads to new policies as feedback to the real system, even with the assistance of artificial intelligence layers (Umeda et al., 2019; Gichane et al., 2020). Considering their functions (Khan et al., 2022) each DT can focus on (i) monitoring a PT, (ii) simulating the future states of a PT, (iii) directly interacting—as an “operational DT”—with a cyber-physical system as PT.

Among the fields of DT application, robotics certainly offers several examples (Girletti et al., 2020; Matulis and Harvey, 2021) of twinning solutions, especially in conditions of HRI like human-robot collaboration (Malik and Bilberg, 2018; Maruyama et al., 2021; Tuli et al., 2021). In particular, literature in robotics offers interesting solutions of intuitive extended reality interfaces (Alfrink and Rossmann, 2019; Burghardt et al., 2020) to ease the interaction of an expert with a DT. In the next section, we propose that such an approach can be further enhanced by emulating certain PT components through a DT and others through a physical replica of the robotic system.

## Phygital Twins in Human-Robot Interaction

Performing holistic, physical, and reality-based interaction with a robotic system is more intuitive for the user than contactless gestures to program or command the device and change its state to accomplish a task (Jacob et al., 2008; Heun et al., 2013; Blackler et al., 2019; Ravichandar et al., 2020). Following this reasoning, we decided to highlight the opportunity of emulating a PT through what we labeled as a “Phygital Twin.” This term has already been used by Sarangi et al. (2018) to describe an IoT setup designed to collect data and represent an environment (even through portable devices) to assist a farmer in precision agriculture paradigms. However, we envisioned the usage of this label for a wider class of solutions by pondering the meaning of the “phygital” attribute outside the domain of twinning processes.

As a neologism (merging two words: physical and digital), this attribute has been typically adopted across various domains like design and marketing, blending real and virtual dimensions as in its etymology (Gaggioli, 2017; Mikheev et al., 2021). This term was used, for instance, to define Tactile User Interfaces (TUIs) like the “phygital map” in Nakazawa and Tokuda (2007), the paradigms of “phygital play” (Lupetti et al., 2015) in mixed reality-based robotic games (MRRGs) (Prattico and Lamberti, 2020), and interactive solutions for work and education proposed during the COVID-19 pandemic (Chaturvedi et al., 2021; Burova et al., 2022).

Overall, these are just examples in a general virtual-real convergence trend (Tao and Zhang, 2017), like cyber-physical twins (Czwick and Anderl, 2020). This trend occurs in healthcare too (Gregory, 2022) about managing chronic conditions and predicting their progress or the therapeutic outcome (Voigt et al., 2021; Barresi et al., 2022). Furthermore, we must highlight how intrinsically phygital are the recent definitions of the metaverse, a digital world embracing cyber-physical systems and also DTs in its connection with the real world (Yoon et al., 2021).

**Figure 1:**
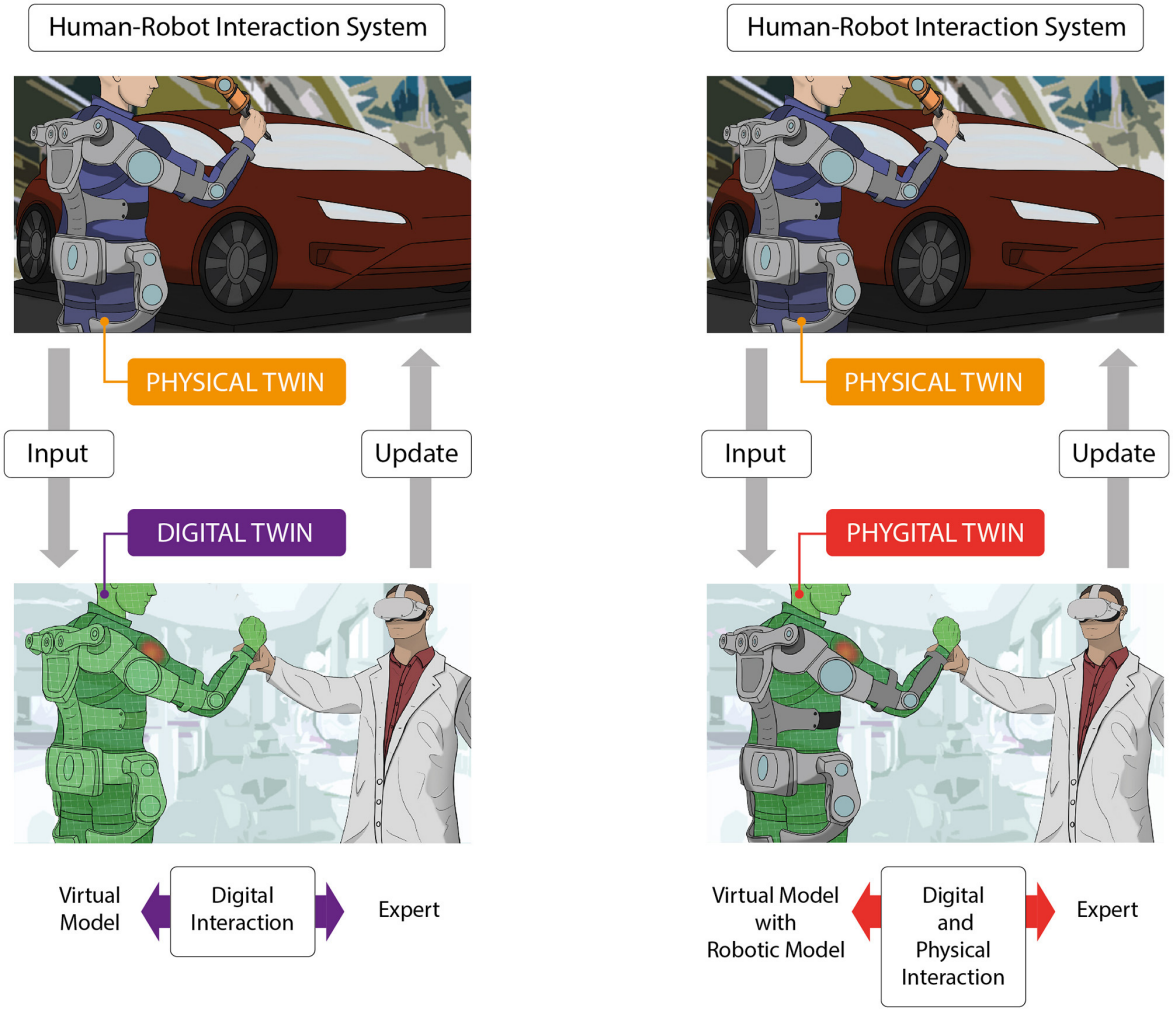
A Physical Twin (PT), based on a human-exoskeleton system in a context of usage, connected (on the left) to its Digital Twin (DT) in green, based on a virtual model of the human-robot interaction (HRI) system, or (on the right) to its Phygital Twin (PDT), based on a holographic human “wearing” a real exoskeleton in a laboratory.

Exploiting the phygital approach we foresee a Phygital Twin (PDT, highlighting both its physical and digital elements) as in the example in Figure 1. Within a PDT, certain components of the PT are replicated by digital objects and others by physical objects within an integrated extended reality model. These physical objects would be secondary instances of the same products (not necessarily a robot) in the PT. In Figure 1, an example of the human-exoskeleton system in a real context is the PT emulated by a DT (in green, on the left), based on a fully virtual model of the HRI system. On the other hand, the same PT can be represented (on the right) by a PDT, based on a virtual human “wearing” a real exoskeleton (identical to the one in the real-world context and, possibly, sustained by a mannequin) into a laboratory. Both settings, visualized by an expert through a mixed reality headset, enable the live visualization of anomalies in the right shoulder of the worker in this example.

Different from the case of the fully virtual model on the left, the expert on the right can decide to alter the phygital model through intuitive physical interactions with the lab exoskeleton (working as a TUI), performing tests according to past and current data from the PT. Indeed, the expert receives visual feedback from the DT and more intuitive visuotactile feedback from the PDT. After obtaining the informed consent of the worker in the PT system, the experts can also update the remote wearable robot software according to their predictions.

Thus, the PDTs enable intuitive phygital interactions with experts to assess and improve the PT. Furthermore, its physical components can emulate the ones of the PT more reliably than a virtual simulacrum because they are based on the same products. The PDT computer-generated elements may also be visualized through a virtual reality headset instead of a mixed reality one, according to the need of depicting the PT context as a whole. However, focusing further on the virtual human component can also be greatly advantageous to deepen our knowledge of the user's conditions, especially in terms of neuromotor and neurocognitive processes, as the next section will propose.

## Neuroergonomic Twinning of HRI Systems

Through digital human modeling (Paul et al., 2021), DTs can contribute to monitoring, assessing, and designing different human-system interactions (Caputo et al., 2019; Greco et al., 2020; Sharotry et al., 2022; Wang B. et al., 2022) according to the perspective of human factors. In particular, neuroergonomics (Mehta and Parasuraman, 2013)—especially computational neuroergonomics (Farahani et al., 2019)—can advantageously exploit twinning for understanding how the human nervous system works in real contexts (Cheng et al., 2022), and improving the design of any item interacting with it. This is certainly true about neuroergonomics in HRI contexts (Cassioli et al., 2021) for applications like monitoring motor control difficulties (Memar and Esfahani, 2018), providing robots with adaptive features (Lim et al., 2021), and improving brain-robot interfaces (Mao et al., 2019). Overall, the exploitation of DTs in this field can inherit the corpus of knowledge in neuroscience, especially when human-machine interactions are investigated (Gaggioli, 2018; Ramos et al., 2021). Interestingly, literature in this area already shows several approaches presenting analogies with PDTs, which can contribute to neuroergonomics in HRI by offering intuitive interactions with a phygital emulation of the human-robot system.

For instance, the field of bionic prosthetics (Frossard and Lloyd, 2021) offers this kind of solution, with emphasis on twinning the residual limb more than the device. Interestingly, Chen et al. (2022) labeled as “mechatronics-twin” a framework integrating a 6-DoF manipulator with biomechanical models to explore, through simulations, the operational behaviors of prosthetic sockets with amputees. Such an example sounds quite close to the concept of PDT, which can have additional features of real-time bidirectionality, intuitive physical interaction, and ecological validity (resemblance with real contexts).

Furthermore, Pizzolato et al. (2019) proposed human neuromusculoskeletal (NMS) system models for DTs to improve the outcome of the interactions between users and assistive or rehabilitative machines. NMS models implemented in robot control solutions can offer phygital features. For instance, the output of the interaction between a user and a mechatronic device (possibly enriched by extended reality solutions) can become a quantifiable index of healthy and pathological conditions and responses to treatments. This would make such an output a peculiar type of digital biomarker (Wright et al., 2017): a “phygital biomarker” or possibly, a “neurophygital biomarker” (a promising step in this direction is based on neuromechanical biomarkers for rehabilomics) (Garro et al., 2021). In line with this reasoning, we could think about “neurophygital twins” to extract biomarkers from the activity of their PTs: mechatronic devices like rehabilitative exoskeletons (Buccelli et al., 2022) or, possibly, any other robot (including humanoids) designed to interact with humans wearing sensors.

Through intuitive phygital interactions between the researcher or the clinician and the lab replica of the same machine in the real world, neuroergonomic hypotheses on psychophysiological and motor processes underlying HRIs can be tested in simulated experiments based on a PDT. We could also envision the development of neurorobotic systems (Li et al., 2019) mimicking neurocognitive and neuromotor processes to physically replace a virtual human model in a PDT: in this case, the neurorobotic model would be validated through its interaction with another machine within the same PDT. However, before addressing such challenges, the current constraints in our knowledge and know-how must be pondered. Besides the technical limitations in twinning (first of all, the computational burden of emulating neural processes in ecologically valid settings, without considering the connectivity issues to approach the real-time standards), we must also highlight how both DTs and PDTs raise ethical issues on privacy and consent in data representation and storage, and on concepts like “normality” and enhancement (Bruynseels et al., 2018; Braun, 2021; Nyholm, 2021). These issues should be discussed within the frame of the enablers and the barriers to twinning adoption (Perno et al., 2020), even pondering the opportunities offered by novel technological frameworks (Yi et al., 2022).

## Conclusion

This position paper presented a novel “twinning design” concept: PDT, based on physical replicas of PT components enriched with virtual models and computational features to establish intuitive and reliable phygital interactions with experts. Thus, a PDT would facilitate the experts' task of assessing and improving the PT conditions. Furthermore, PDTs provide neuroergonomics with tools for iterative human-centered design and evaluation of robotic systems into a “metalaboratory” before and after their deployment.

## Author Contributions

GB devised the conceptual contents and structure of the paper and wrote the initial draft. CP, ML, and LDM improved the document and considering further potential applications of the proposed approach. All authors revised and approved the manuscript.

## Conflict of Interest

The authors declare that the research was conducted in the absence of any commercial or financial relationships that could be construed as a potential conflict of interest.

## Publisher's Note

All claims expressed in this article are solely those of the authors and do not necessarily represent those of their affiliated organizations, or those of the publisher, the editors and the reviewers. Any product that may be evaluated in this article, or claim that may be made by its manufacturer, is not guaranteed or endorsed by the publisher.
